# Microneedling in facial recalcitrant melasma: report of a series of 22 cases*

**DOI:** 10.1590/abd1806-4841.20154748

**Published:** 2015

**Authors:** Emerson de Andrade Lima

**Affiliations:** 1 Santa Casa de Misericórdia do Recife - Recife (PE), Brazil

**Keywords:** Bleaching agents, Face, Melanins, Melanosis, Treatment failure

## Abstract

Melasma is a chronic skin disorder that results in symmetrical, blotchy, brownish facial pigmentation. It is more common in women than in men, it generally starts between 20 and 40 years, and it can lead to considerable embarrassment and distress. The aims of this article is to evaluate the treatment with the microneedling method in 18 female and 4 male with recalcitrant melasma. All patients demonstrated good results. In conclusion, microneedles appears to be a promising therapeutic method for melasma.

Melasma is a common hypermelanosis, characterized by macules with irregular borders, with intensity from light to dark brown, located in photo-exposed areas and primarily affecting women of childbearing age. Three clinical presentations of melasma were identified based on histopathologic findings: epidermal melasma, when the pigment is deposited in the basal and suprabasal layer; dermal melasma, when melanophages filled with melanin are found in the superficial and middle dermis; mixed melasma, when findings of the two previous types of melasma are present.^1,2^ The examination in Wood's light contributes to differentiate the epidermal from dermal melasma in types I-IV of Fitzpatrick scale.^1^ Considering its recalcitrant characteristics, treatment can rarely keep the individual free of melasma for a long time, despite the many proposals available.^3-4^ More recently, it was proposed the application of active medications by piercing the skin with needles: the microneedling.^5,6^ To this end, a polyethylene roll wedged by stainless and sterile steel needles, symmetrically aligned in rows, totaling 190 units, performs back and forth movements guided by a uniform pattern of petechiae.^7^ Lima et al (2013) proposed a classification relating the length of the needle of the devices used with depth of predicted damage.^8^

This article presents the evaluation of the medical records of 22 patients with recalcitrant melasma - that is, unresponsive to topical lightening and sunscreen - treated by microneedling, following the same protocol and performed by the same doctor from January 2012 to January 2015 (Table 1). Patients were clinically diagnosed with melasma and had the examination confirmed by Wood's light and dermatoscope. Photographic documentation was performed by the same investigator and with the same digital camera immediately before the procedure and after 2 months. Individual use of microneedling without the use of any active topic medication during treatment was established as therapy protocol. The procedure was performed under topical anesthesia with 4% lidocaine cream (Dermomax^®^) applied 30 minutes before the intervention. An instrument with needles length of 1.5 mm was used (Dr.Roller^®^ Mooham Enterprise Co. Gyeonggi-do South Korea, nº ANVISA 80669600001). The treatment proceeded with back and forth movements, approximately 10 times in 4 directions, drawing four bands that overlapped, resulting in a diffuse erythema and discrete punctuated bleeding. After 24 hours and in the days that followed patients were instructed to use at night an industrialized depigmentation formula (0.05% tretinoin + 4% hydroquinone + 1% fluocinolone acetonide) and industrialized tinted sunscreen with SPF 60. The same procedure was carried out 30 days after the first treatment.

**Table 1:** Characteristics of the evaluated group

	Number of patients (%)
**Gender**	
Female	18 (82)
Male	4 (18)
**Age (years)**	
22-30	12 (55)
31-40	8 (36)
>40	2 (9)
**According to Fitzpatrick scale**	
II	4 (18)
III	10 (45)
IV	8 (36)
**Time of onset of melasma (years)**	
< 5	4 (18)
5-10	10 (45)
11-20	8 (36)
**Uninterrupted use of skin lightening (years) <1**	0 (0)
1-5	12 (55)
6-10	10 (45)

One hundred percent of patients reported satisfaction with the results. The degree of discomfort during treatment was considered well tolerated by 16 (70%) patients and 6 (30%) of them informed they didn't feel any pain. All patients reported having returned to their activities immediately after the procedure. The authors considered the results from good to very good on a scale of very good, good, reasonable and poor. Authors also found that all 22 patients were responsive to the technique used and that they would repeat the same procedure in other cases with similar indication. Eleven of the evaluated patients are already at 24 month follow-up after the first procedure, and they have been maintaining skin lightening similar to that observed with two months (Figure 1).

**Figure 1:**
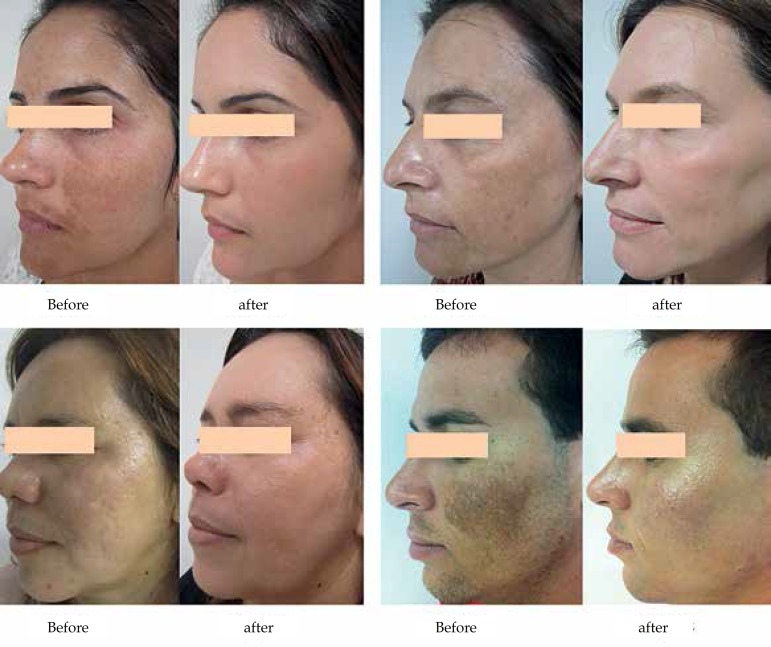
Patients before and after 60 days of treatment

Despite the wide therapeutic arsenal available for the treatment of melasma, including new to old active topical medications, technologies with lights and peelings, clinical control of this melanodermia is extremely challenging. The proposed application of active medication with depigmentation action has been used, but little is said about the isolated action of microneedling with potential lightening effect.^8-10^ The author's observation during five years of cases treated for photodamage and acne scars that showed substantial skin lightening led to the use of the procedure for the treatment of patients with recalcitrant melasma. In this retrospective analysis of 22 patients it can be assumed that the substantial lightening observed in the whole group was achieved by modifications occurred in the skin after moderate injury caused by needles.^8^ The physiogenesis process remains unclear, but the experience of the authors demonstrates satisfactory and reproducible results. Therefore the authors conclude:

1. Microneedling alone, with 1.5 mm needle length, without the addition of any active medication, can cause lightening of skin stains in patients with recalcitrant melasma.
2. Trauma caused in the procedure must be modest and the use of skin lightening and sunscreen following the procedure becomes mandatory.
3. Although some theories proposed, the exact mechanism of skin lightening is not yet well established.
4. New controlled studies are required in order to clarify the mechanism of action of microneedling on melasma, but it's possible to conclude that the evaluated group showed promising results with this new therapeutic proposal.
